# Towards advance care planning in pediatrics: a qualitative study on envisioning the future as parents of a seriously ill child

**DOI:** 10.1007/s00431-020-03627-2

**Published:** 2020-03-19

**Authors:** Jurrianne C. Fahner, Thessa W. Thölking, Judith A. C. Rietjens, Agnes van der Heide, Johannes J. M. van Delden, Marijke C. Kars

**Affiliations:** 1 Julius Center for Health Sciences and Primary Care, Medical Humanities, University Medical Center Utrecht, Internal mail no Str. 6.131, P.O. Box 85500, 3508 GA Utrecht, The Netherlands; 2 Department of Public Health, Erasmus Medical Center, Rotterdam, The Netherlands

**Keywords:** Advance care planning, Communication, Pediatric palliative care, Shared decision making

## Abstract

Advance care planning enables parents to discuss goals and preferences for future care and treatment of their seriously ill child. Although clinicians report parental factors as common barriers for advance care planning, parental views on reflecting on their child’s future have had limited exploration. A clear understanding of their perspectives might help clinicians to implement advance care planning tailored to parental needs. This interpretive qualitative study using thematic analysis aims to identify how parents envision the future when caring for their seriously ill child. Single interviews and two focus groups were attended by 20 parents of 17 seriously ill children. Parents reported to focus on the near future of their child. However, their actions and deeper thoughts showed perspectives towards a further future. Future perspectives initial focused on practical, disease-related themes, but more existential elaborations, reflecting underlying life values, were also identified. Parents needed acknowledgement of their challenging situation, care tasks, and expertise as a precondition for sharing their deepest thoughts regarding the future of their child.

*Conclusion*: When envisioning the future of their seriously ill child, parents tend to stay in the near future, whereas they value the opportunity to share further thoughts within a compassionate relationship with clinicians.

**Table:** 

**What is Known:** • *Parents prefer open and honest information about their child’s illness and prognosis and they value the concept of advance care planning, while they emphasize the need for an individualized approach.* • *Health care professionals see parental factors like unease and emotional burden as key barriers for advance care planning.*	
**What is New:** • *When envisioning the future of their seriously ill child, parents tended to stay close to the near future initially, with a focus on disease-related, practical themes. Ongoing conversations uncovered deeper, value-based elaborations towards the future. To engage parents in advance care planning, the future needs to be discussed in relation to the present and the past.* • *There is “no sharing without caring”. Parents who felt cared for and acknowledged in their challenging context by clinicians, were open to share their perspectives on the future of their seriously ill child. To share deeper motives and values underlying goals and preferences for future care and treatment, parents need a stimulating attitude of listening and encouragement from clinicians to express their feelings.*

**Electronic supplementary material:**

The online version of this article (10.1007/s00431-020-03627-2) contains supplementary material, which is available to authorized users.

## Introduction

As survival rates have improved in pediatric care due to medical and technological advances, the number of children and young adults living with life-limiting conditions has increased over time [11].

These children and their families are in need of palliative care and often need support in decision making about future treatment and care [10]. However, early integration of palliative care discussions remains challenging. A qualitative interview study from Hungary showed that physicians tend to place palliative care at the end of a disease trajectory, when there are no curative options left [22]. Advance care planning (ACP) aims to facilitate early planning of future treatment and care, including end-of-life care, through exploration and understanding of individual values, preferences, and goals for care and treatment [23].

Although research on pediatric ACP is still in its infancy, emerging evidence suggests that families and clinicians value the concept of ACP, even earlier in disease trajectories than is customary practice [5, 15, 17–21].

However, it has also been established that parents, whilst valuing ACP greatly, simultaneously experience ACP as emotional [19]. Clinicians’ receptiveness to parental feelings of unease poses a barrier to initiate ACP conversations with parents [1, 7, 18, 24]. Consequently, although both parents and clinicians contemplate future care, a substantial exchange of their perspectives does not seem to occur sufficiently [7, 13, 24]. So far, current literature focuses on the experiences of bereaved parents, mainly within the end-of-life phase [5, 19].

To engage parents and medical teams in ACP, clinicians need a profound understanding of parental preferences towards anticipating and discussing the future. Insight into parental experiences and perspectives regarding the future, both early and late in a disease trajectory, is lacking. Therefore, this study aims to elucidate how parents of children with life-limiting conditions contemplate the future and under which conditions parents share these future perspectives with clinicians caring for their child.

## Methods

To elucidate parents’ perspectives on contemplating the future, we conducted an interpretative qualitative interview study using an inductive thematic analysis [2, 4, 26]. The COmprehensive consolidated criteria for REporting Qualitative research (COREQ) were used to structure the study report [27].

### Sample

A purposive sample of Dutch-speaking parents of children diagnosed with a life-limiting condition under 18 years of age was included. To capture a wide range of perspectives, variation was sought with respect to the parent’s gender and education, the child’s diagnosis, stage of illness, life expectancy, and age. Both bereaved and non-bereaved parents were eligible. Bereaved parents were included as they are able to reflect on their thoughts about their child’s future in retrospect, while overseeing their child’s whole disease trajectory, including end-of-life. Pediatricians in one university medical center and two peer supporters introduced the study to parents and asked permission for the researchers to contact them.

### Data collection

Parents were offered a choice to participate in a prescheduled focus group interview or an individual face-to-face interview. The interviews took place between June 2018 and March 2019. Individual interviews were scheduled at a location and time as preferred by the parents. The two focus group interviews were moderated by JF (trained qualitative researcher, MD) and MCK (experienced qualitative researcher, RN). JF conducted the individual interviews. The interviews were guided by a topic list, which was based on literature and expert knowledge. The topics included future time frame, future perspectives, sharing of future perspectives, future goal setting and decision making. (Online Resource 1) Interviews were audio recorded and transcribed verbatim. Parent and child demographic variables were collected through an additional questionnaire. The research ethics committee of the University Medical Center Utrecht determined that this study was exempt from the Medical Research Involving Humans Act (September 27, 2017; Reference number: 17–662/C). All participants provided written informed consent.

### Data analysis

A thematic analysis was performed [2]. During the entire process, three researchers (JF, TT, MCK) were involved. Researcher triangulation was ensured to improve reliability and validity of the analysis. The thematic analysis consisted of three phases [4, 26]. First, the core researchers (JF, TT, MCK) individually (re)read the transcripts of five individual interviews to get familiar with common aspects and phrases. Two researchers (JF, TT) individually analyzed and coded meaningful fragments in the light of the research question and compared interpretations together. The meaning of the separate text fragments was determined by interpreting them in light of the whole interview [16]. Initial codes were recoded, resulting in an adapted code list with themes and concepts at a more conceptual level [2]. During the second phase, new interviews were read and discussed by two researchers (JF, TT). One researcher (JF) coded all transcripts, supported by the software program Nvivo 11. The code tree was evaluated and adjusted. Lastly, the research team (JF, TT, MCK) identified key themes and related subthemes. The researchers went back and forth between the different steps to guarantee constant comparison. Code saturation was reached on a conceptual level [14].

## Results

In total, 20 parents of 17 children were interviewed. Ten parents attended a focus group interview of five participants each. Individual interviews took place in the hospital (*n* = 8), at home (*n* = 1) and at the parent’s workplace (*n* = 1). The interviews lasted from 30 min to 3 h. For respondent characteristics, see Table 1.

**Table 1:** Parent and child characteristics

	*n* (%)
Parent characteristics (*n* = 20)	
Female	15 (75)
Age	
30–40 years	9 (45)
40–50 years	8 (40)
> 50 years	3 (15)
Marital stage	
Married/cohabiting	18 (90)
Not cohabiting	2 (10)
Caucasian race	20 (100)
Level of education	
Secondary school	1 (5)
Vocational education	4 (20)
High school	6 (30)
University	9 (45)
Religion	
Protestant	11 (55)
None	9 (45)
Child characteristics (*n* = 17)	
Female	5 (30)
Deceased	
Total	6 (35)
< 2000	1 (17)
2000–2010	1 (17)
> 2010	4 (67)
Age at death/at interview	
< 1 year	3 (18)
1–5 years	6 (35)
5–12 years	5 (29)
> 12 years	3 (18)
Diagnosis	
Chromosomal anomaly	7 (41)
Congenital heart disease	4 (24)
CNS tumor	2 (12)
Cystic fibrosis	1 (6)
Neuromuscular disease	1 (6)
Epilepsy syndrome	1 (6)
Perinatal asphyxia	1 (6)
Age at diagnosis	
< 1 year	12 (71)
1–5 years	3 (18)
> 5 years	2 (12)

### Attitudes towards the future

All parents expressed some thoughts about the future of their child and family. Several triggers stimulated them to contemplate the future. These were often disease-related triggers, like upcoming medical evaluations, procedures, or decision making. Besides that, questions about the child’s development in the context of its disease stimulated parents to think about the future. Triggers could also be related to safeguarding the continuity of care. Parents reported external triggers, like changes in laws and financial support, and internal triggers, such as worries about the long-term task of caregiving and related parental burden of care. Lastly, parents mentioned that existential questions stimulated them to think about the future of their child. These questions could arise from prior experiences with illness, death, and dying or from their spiritual beliefs. These questions made parents think about their underlying values and influence of these values on future decision making.

Four main themes were identified when parents were asked to envision the future of their child. It was seen that (1) there is a focus on the near future; (2) future perspectives are intertwined with experiences in the present and the past; (3) future perspectives range from a disease-related orientation to a value-based orientation; and (4) there is “no sharing without caring.” Representative quotations were chosen to illustrate the identified themes. Perspectives on the future while caring for a seriously ill child as described below were quite similar for both bereaved and non-bereaved parents (Table 2).

**Table 2:** Quotes that illustrate parental attitudes towards the future and sharing of future perspectives

(sub)Theme	Quote
Focus on the near future	
1A Initial orientation on the near future	R20: mother of a girl, 6 years, MD. *“Our live was really divided into periods until the next MRI. I could not look further than the next scan, no way. I got angry or anxious when we got invitations for events scheduled after that period.”*
1B Preparatory actions show further perspectives	R3: mother of a boy, 3 months, NMD. *“Not to prepare everything in detail, but I bought clothes for him to wear in the coffin, you know?... And then I put them away in a bag over there.”*
Intertwinement of future perspectives with experiences from the present and the past	
2A Future perspectives are related to the current situation	R7: father of a boy, 4 years, NDM. *“He already survived his own prognosis. We are going to help him stay the longest-living infant with this syndrome.”*
2B Prognostic certainty stimulates thinking about worst-case scenarios further away	R13: mother of a girl, 1 year, NMD. *“The doctors are just really sad about her future. We distinctly discussed how we will…. what we will do when she loses consciousness [during an event at home]. Shall we call the doctor, or will take her in our arms, where she will pass away?”*
2C Future perspectives are related to experiences from the past	R6: mother of a boy, 4 years, NMD. *“We proved with our other child [parents lost another child with the same diagnosis after withdrawal of life sustaining treatment], grimly said, that we are capable of taking a child off the ventilator. That somehow grants you the confidence that, even though you never thought you would be capable of doing that, you might be able to do it again.”*
2D Prior decision making influences attitude towards the future	R1: mother of a boy, 1 year, NMD. *“If we […] would have known everything, that it would be so tough, we would have […] not carried to term. In hindsight. But at that time you did not know. But it is so beautiful to know him. You would not have known that it could be so beautiful.. So he keeps you going… there is nothing else to do..*
2E Life views connect past, present and future	R13: mother of a girl, 1 year, NMD. *“This we really know…that eventually her life is simply in God’s hands and He knows. He knew her beginning and He knows her end, her life’s end. And we hope it [her life] will not end sometime soon.”*
Future perspectives range from a disease-related orientation to a value-based orientation	
3A An initial practical, disease-related orientation	R11: mother of a boy, 6 years, NDM. *“On the one hand there is this question: ‘how long will his future be?’ and on the other hand ‘how are we going to fulfill his care needs?”*
3B More existential thoughts emerge in deeper conversations	R13: mother of a girl, 1 year, NMD. *“Yes, I would really love to see a little bit of development, just a little bit of interaction [with her daughter], but actually I do not really hope for it anymore, because I do not believe it will happen. It is more like a wish.”*
3C Defining future goals of care needs deliberation	R11: mother of a boy, 6 years, NDM. *“Uhm, well… Look, in the ideal situation we would prevent big problems, more big problems, in the future. But if you are talking about cure [as opposed to care], this is a difficult thing, because you cannot foresee what will cross your path in the future.”*
3D Discussing treatment limitations touches underlying values	R5: mother of a boy, 3 years, NMD. *‘Unjustly, the question whether it has been enough or whether we should continue treatment is asked about him very often…Other children are very ill as well and sometimes unhappy, but no one dares to ask this question in their case…While with [her son] it is asked all the time… That is quite confrontational… very painful…. (R5)*
No sharing without caring	
4A Need for acknowledgment challenging parental context	R10: father of a girl, 7 years, NMD. *“I am always feeling ill and on the move, and you can just see that I will not make it. You can see how my engine is starting to fail…”(R10)*
4B Need for acknowledgment growing parental expertise	R7: father of a boy, 4 years, NMD. “*When I call the neurologist to say it is not OK with my son’s epilepsy, than he will take some action. He will not ask any further questions, but trusts me in my observation the epilepsy is getting worse and something has to be done about it.*
4C Attention to perspectives outside the medical domain	R5: mother of a boy, 3 years, NMD. *“I would appreciate it [to discuss matters out of the medical domain]. His emotional wellbeing and his development are part of who he is.*
4D Awareness of the child’s identity	R5: mother of a boy, 3 years, NMD. “*He is not just a respiratory infection, he is simply a human being.”*
4E Need for consistency towards shared care goals	R2: mother of a boy, 3 months, NMD. *“I believe that as long as the shared goal is being put forward, you are already halfway there. Then you’ll have an understanding of each other [parents and clinicians], respect each other and appreciate each other deeply.”*

*MD* malignant disease, *MRI* magnetic resonance imaging, *NMD* non-malignant disease

### Focus on the near future

Although many parents said to live one day at a time, they could not neglect future perspectives. As parents expressed thoughts about the future of their child and family, they focused on the near future initially. They felt being withheld from looking further ahead by recurrent episodes of clinical deterioration of their child, prognostic uncertainty, upcoming medical procedures, and the actual burden of daily care giving (Table 2, Quote 1A). Although most parents limited their reflections to the near future initially, they showed contemplation of a further future in actions they reported. These actions showed that parents prepare themselves, at least in a practical way, for a further future where deterioration of their child’s condition might occur. For example, these were practical arrangements for the child’s death (Table 2, Quote 1B) and integration of certain facilities in a rebuilding plan for their homes.

### Intertwinement of future perspectives with experiences in the present and the past

When parents shared perspectives about the future in the interviews, it was seen that these perspectives were very intertwined with experiences in the present and the past. First, the content of their future perspectives was influenced by their attitude towards the current situation. Parents who were suffering and struggling in the present, tended to see the future as a black box, while parents with a consistent, balanced view on the actual situation of their child could more easily look forward. This did not seem to be related to an either better or worse prognosis (Table 2, Quote 2A). Besides that, in case of experiencing more prognostic certainty in the present, either better or worse, parents showed more ability to elaborate on the future. If future scenario’s seemed realistic to parents, they were more tempted to reflect on those situations, even though it confronted them with unfavorable outcomes for their child (Table 2, Quote 2B). Some parents mentioned that feeling at peace with intense end-of-life experiences in the past, made them more open-minded to think and discuss about a future where similar scenarios could occur (Table 2, Quote 2C). Few parents envisioned the future in relation to decisions made in the past. This made them think about the life they could have had as a family, if only they had made different choices in the past. These elaborations were followed by thoughts about all the good things being a parent of their seriously ill child had brought them. These positive thoughts supported them to face the future (Table 2, Quote 2D). Some parents experienced a connection between past, present and future based on their life views and spiritual beliefs. They framed their perspectives on the future as part of a continuing life story, influenced by a higher power, like God (Table 2, Quote 2E).

### Future perspectives range from a disease-related orientation to a values-based orientation

Most parents mentioned practical, disease-related perspectives at first, when asked about their views on the future. Common topics were disease progression, next medical evaluations, the child’s development, financing the care costs, safe-guarding care at home, maintaining family life and organization of multidisciplinary care (Table 2, Quote 3A). When asked about their thoughts on the future, most parents did not talk spontaneously about underlying life views, values, hopes, fears, and worries. However, when specifically asked about, they presented all sorts of reflections on more existential themes. Hopes for the future could be concrete, realistic hopes or wishes and dreams, that were to be cherished (Table 2, Quote 3B). Fears and worries regarding the future concerned the loss of their child to death, facing difficult decisions, possible suffering of the child, the ongoing heavy burden of care, and achieving a life as normal as possible for their child. Some parents expressed that addressing these fears was emotional and burdensome to them. Recognizing or discussing their fears confronted parents with worst-case scenarios as a reality and disrupted their coping strategy of focusing on the here and now. However, parents demonstrated this made them not unwilling to contemplate the future. It enabled them to prevent or prepare themselves for a feared situation and left them with a greater peace of mind in the present. Some parents mentioned in hindsight they would have valued more attention to their fears, because they felt overwhelmed and unprepared when a worst-case scenario occurred.

When parents were asked about future care goals for their child, a distinction between disease-related aims and value-based aims was seen as well. Some parents had clear short-term disease-related aims, such as correction of a tracheostomy. These parents could more easily formulate goals of future care. Parents who reported broader, all-encompassing, value-based aims for their child, such as being happy or trying to live an ordinary life, had more difficulties to demonstrate how these aims could guide them to formulate goals of future care (Table 2, Quote 3C). Some parents mentioned that taking the perspective of their child, like “what would my child value the most,” helped them to define goals of future care and treatment.

Most parents recalled discussions about treatment limitations when thinking about future goals of care. They showed to experience these discussions as touching their underlying values, whereas clinicians framed these discussions more in the context of the child’s disease and any medical futility (Table 2, Quote 3D). Some parents addressed treatment limitations themselves because they considered this an essential part of what they valued as good care for their child. However, parents emphasized they would prefer clinicians to initiate these discussions, because the accompanying emotional distress could be a parental barrier to initiate a conversation about treatment limitations.

### No sharing without caring

Although all parents presented elaborate thoughts about the future of their child during the interviews, few said to have discussed the rich content of these thoughts with their clinicians. Several factors were identified that would support sharing of future perspectives with clinicians. First, parents mentioned they need acknowledgment of their challenging context. Parents expressed they felt clinicians have no idea of the impact of caring for a seriously ill child on their daily life. They showed a need for acknowledgement of the burden of care that is on their shoulders (Table 2, Quote 4A). Second, parents want their growing expertise to be acknowledged and be taken into account when it comes to medical decision making (Table 2, Quote 4B). Most parents felt a struggle to be treated as the expert of their child. Some parents felt being judged for their perspectives on their child’s future and feared unintended consequences for their child’s care, without opportunities for reconsideration. Third, parents reported little room to share perspectives outside the medical domain, although they would appreciate it (Table 2, Quote 4C). Besides that, parents expressed to value clinician’s awareness of the child’s identity apart from his or her disease (Table 2, Quote 4D). When their child was seen as an individual person, with an own life story, they felt sharing perspectives on their child’s future with clinicians made more sense.

Lastly, parents expressed a need for a consistent approach of clinicians regarding future care and treatment over time and among different disciplines. Parents reported to struggle to get all clinicians on the same page. If parents felt a shared goal within the team and felt part of the team, this positively influenced their openness to share their perspectives (Table 2, Quote 4E).

## Discussion

When envisioning the future of their seriously ill child, parents tended to stay close to the here and now. However, parents showed to experience thoughts that go beyond the present, even beyond their child’s death, and they reported activities showing preparations for a further future. When sharing future perspectives, parents focused on practical and disease-related themes initially. More existential, value-based perspectives were shared less spontaneously, mostly after being specifically asked about. However, parents reported to value opportunities to share their deepest thoughts with clinicians. When parents experienced a relationship of trust and reciprocity with their clinician and felt acknowledged as experts of their child, they shared more elaborate thoughts about the future with their clinician.

Sharing of preferences and goals for future care is a key element of ACP. The main findings of our study provide some insights that might be useful for the further development and implementation of pediatric ACP. First, whereas ACP aims to discuss future situations, parents might need a stepwise approach that begins close to their actual situation. With the current tendency to initiate ACP early in a disease trajectory [23], aiming to oversee a future which is further away, it becomes even more important to achieve a shared understanding of the child’s illness and the actual situation as a first step in ACP [3, 6, 9]. This need for an initial focus on disease-related issues when discussing the future is in line with earlier research, where the strive for controlled symptoms and controlled disease was the key parental aim [28]. Another study identified taking control as one out of four coping strategies of parents who take care of their child receiving palliative care and found that taking control reduced emotional distress [29]. In our study, parents who showed to feel in control over their daily live and care tasks seemed to be able to overview the future more easily, whereas parents who were struggling in their parenting role had more difficulties to achieve a thorough perspective on the future. Our study suggests that sharing of future perspectives in the context of the actual situation supports parents to identify what really matters to them and where they should focus on together with the clinical team. Consequently, sharing these thoughts with clinicians showed to support parents in pursuing their goals and meeting their needs [5, 19].

Second, parents in our study reported that they did not naturally share their more existential thoughts with clinicians. Contemplating more sensitive issues regarding the future, like hopes, fears, and worries, is a demanding and sometimes burdensome endeavor to parents [19]. However, this parental unease does not reflect unwillingness to talk about these issues. Therefore, it should not be seen as a barrier for ACP, although clinicians tend to do so [7]. Findings from our study indicate that parents might not experience sufficient opportunities from clinicians to share their deepest thoughts regarding the future. Whereas ACP includes the physical, psychological, social, and spiritual domain [25], parents might not expect clinicians to show interest in all these domains. In that way, both parents and clinicians continue to focus on medical issues, leaving other domains undisclosed. This might complicate a shared understanding of future care goals and hinder shared decision making. Conversation guidelines may help clinicians to address existential issues in the context of ACP [9, 30].

Third, in line with earlier research, the results of this study underline the importance of a trustful relationship between parents and clinicians when sharing future perspectives [12, 19]. Our study adds that parents need to feel cared for as a precondition to share future perspectives. This applies in particular to sharing of deeper, personal perspectives. It is known that parents have mixed experiences in their relationships with clinicians [29]. Parents in our study showed clear factors that influence this relationship positively. Clinicians who take these factors into account when discussing the future with parents, might create more openness and deeper insight in parental preferences and underlying values. Ongoing research continues to report that key barriers for ACP as perceived by clinicians, are, in their perspective, related to parental factors [8]. Our study illustrates that those perceived barriers need to be approached from a different point of view. Parents may indeed face challenges when thinking about and sharing future perspectives, but they value attention to their deepest fears and worries, and can reflect on what they need in sharing future perspectives. These insights can be helpful for clinicians to approach parents in an appropriate way, instead of refrain from ACP, based on perceived parent-related barriers.

This study had some strengths and limitations. Our study included both non-bereaved and bereaved parents, whereas research in this field is often based on experiences of bereaved parents alone [5, 19]. We considered both perspectives valuable. Non-bereaved parents share their current experiences, while actual facing a challenging future. However, their current coping strategies might influence their perspectives [29]. Bereaved parents can reflect on their child’s end of life. Despite the influence of recall bias and coping with bereavement, they can reflect on what they wish that could have gone differently. Our study did not focus primarily on experiences with ACP itself, as has been studied before [5, 19], but focused on how parents envision the future when caring for a seriously ill child and on their attitude regarding sharing of future perspectives with others. This knowledge might support further research to develop strategies to implement ACP in pediatrics and align ACP to parental needs. Our findings might be limited by the diversity of interview settings. Some parents were interviewed during admission of their child, which might have influenced their perspectives. A shorter duration of some interviews, due to other appointments of the respondents, might have caused parents to refrain from exposing their vulnerability through complete openness. However, this might be a reflection of daily practice, were all kind of actualities effect conversations about future care. Other limitations were the recruitment of some parents by peer supporters and the predominantly participation of highly educated mothers, which may have biased the results.

## Conclusion

All parents in our study contemplated the future to varying degrees of extent, with a primary focus on the near future. However, exploration of deeper thoughts and occurrence of preparatory actions revealed a scope to a further future. Future perspectives are intertwined with experiences in the present and the past. Sharing perspectives towards the future within a trustful relationship between parents and clinicians can give deeper insight in family values, preferences, and goals for future care.

## Electronic supplementary material

ESM 1 (DOCX 19 kb)

## Abbreviations

ACP: Advance care planning
