# Supplementary material for: Towards advance care planning in pediatrics: a qualitative study on envisioning the future as parents of a seriously ill child
Source: Eur J Pediatr. 2020 Mar 19;179(9):1461–8. doi: 10.1007/s00431-020-03627-2 (PMC7413894; doi:10.1007/s00431-020-03627-2)
Supplement: Supplementary file 1 — (DOCX 19 kb) [file 431_2020_3627_MOESM1_ESM.docx]

**Towards advance care planning in pediatrics: a qualitative study on envisioning the future as parents of a seriously ill child**

Jurrianne C. Fahner^a^, Thessa W. Thölking^a^, Judith A.C. Rietjens^b^, Agnes van der Heide^b^, Johannes J.M. van Delden^a^, Marijke C. Kars^a^

**Affiliations:** ^a^Julius Center for Health Sciences and Primary Care, University Medical Center Utrecht, Utrecht, the Netherlands; and ^b^Department of Public Health, Erasmus Medical Center, Rotterdam, the Netherlands

**SUPPLEMENTS**

Supplement 1 – Topic list

**Supplement 1. Topic list interviews**

| Future | - Do/did you look forward to your child’s future?  - When you look/looked forward to the future of your child, what thoughts come/came to your mind?  - What do/did you consider to be important when thinking about your child’s future? |
| --- | --- |
| Sharing | - Do/did you talk with your pediatrician about your child’s future? Or with other clinicians?  - Which topics do/did you talk about? Where there any topics you could not discuss about with your child’s clinicians?  - Do/did you talk about your child’s prognosis? How is/was that feeling for you?  - Do/did you talk about what is important to you when thinking about your child’s future?  - Do/did you talk about future scenario’s regarding your child’s condition?  - Would you like to see anything different in conversations with clinicians? |
| Specific topics | - How do/did you think about your hopes for your child? How do you value talking about hopes?  - Do/did you talk about your fears and worries?  - Do/did you have any experiences with treatment limitations? What experiences? |
| Decision making | - What is/was your role in decision making regarding your child’s future care and treatment? How do/did your perspectives on the future play a role in there? |
| Goals | - What future goals do/did you aim for with your child?  - Is/was there anything you certainly did not want for your child’s future? |
